# Preparation and Properties of Poly(butylene adipate-co-terephthalate)/thermoplastic Hydroxypropyl Starch Composite Films Reinforced with Nano-Silica

**DOI:** 10.3390/polym15092026

**Published:** 2023-04-24

**Authors:** Zehao Li, Hui Li, Muxi Wang, Zhongyan Zhang, Liting Yang, Lijun Ma, Hong Liu

**Affiliations:** 1 School of Chemistry, South China Normal University, Guangzhou 510006, China; 2020022533@m.scnu.edu.cn (Z.L.);; 2 Key Laboratory of Theoretical Chemistry of Environment Ministry of Education, South China Normal University, Guangzhou 510631, China; 3 State Key Laboratory of Advanced Processing and Recycling of Non-Ferrous Metal, School of Material Science and Engineer, Lanzhou University of Technology, Lanzhou 730050, China; lihui0226@163.com; 4 School of Environment, South China Normal University, Guangzhou 510006, China; 5 Key Laboratory of Analytical Chemistry for Biomedicine, South China Normal University, Guangzhou 510006, China

**Keywords:** PBAT, thermoplastic hydroxypropyl starch, nano-silica, reinforcement

## Abstract

The use of biodegradable plastics is gradually increasing, but its expensive cost limits promotion. In this study, poly(butylene adipate-co-terephthalate)/thermoplastic hydroxypropyl starch reinforced with nano-silica (PBAT/TPHS_g-SiO2_) composite films with high hydroxypropyl starch content were prepared in a two-step process. The effect of reinforced thermoplastic hydroxypropyl starch on the mechanical, thermal, processing properties, and micromorphology of the composite films was investigated. The results showed that the tensile strength of the composite films was significantly improved by the addition of nano-silica, with 35% increase in horizontal tensile strength and 21% increase in vertical tensile strength after the addition of 4 phr of nano-silica. When the content of thermoplastic hydroxypropyl starch reinforced with nano-silica (TPHS_g-4SiO2_) is 40%, the horizontal and vertical tensile strengths of the films are 9.82 and 12.09 MPa, respectively, and the elongation at break of the films is over 500%. Electron micrographs show that TPHS_g-4SiO2_ is better homogeneously dispersed in the PBAT and exhibits a bi-continuous phase structure at a TPHS_g-4SiO2_ content of 40%. In this study, the blowing PBAT/TPHS_g-4SiO2_ composite films effectively reduce the cost and still show better mechanical properties, which are suitable for packaging applications.

## 1. Introduction

With the increasing awareness of environmental protection, biodegradable plastics have received a great deal of attention. PBAT has been the most widely used petroleum-based fully biodegradable polyester so far and has similar processing and usability to low density polyethylene (LDPE) [1]. It has received widespread attention for applications in the packaging industry, biomedical, industrial, and agricultural sectors [2,3,4]. Due to the high production costs of PBAT products, difficulties have been encountered in large-scale commercial applications. Reducing the manufacturing cost of PBAT packaging materials has become the most important factor affecting the widespread use of PBAT-based materials. Blending PBAT with inexpensive inorganic non-metallic powders [5,6,7,8] or naturally degradable polymers such as starch [9,10,11,12,13,14], lignin [15,16], bamboo flour [17], and corn marmalade [18] was an important way to prepare low-cost PBAT-based composites.

Starch was considered to be one of the most promising fillers for PBAT-based composites due to its abundant source, low cost, renewable, and fully biodegradable characteristics [19,20]. It reduced the cost of PBAT materials without compromising the biodegradable characteristics of the composite. However, the melting temperature of starch is much higher than its decomposition temperature [21,22] and the poor compatibility of starch with PBAT made direct blending of high levels of starch with PBAT unfeasible. The starch could only be mixed with biodegradable polymers after it has been turned into plasticized starch [9,23,24,25,26,27,28].

A number of studies have focused on improving the properties of PBAT/TPS composite films, particularly the mechanical strength, by improving the compatibility between PBAT and TPS, or by adding reinforcement directly to the blending system. Table 1 lists the results of this year’s research on PBAT/TPS composite films. As can be seen from the results of these studies, although the compatibilizers were effective in improving the compatibility between the two phases of PBAT and TPS, a large part of these did not significantly increase the mechanical strength of composite materials.

The reasons for this may be that the mechanical properties of thermoplastic starch are poor, which has a mechanical strength of 1.0–5.0 MPa and a high-water absorption capacity [29,30,31]. Therefore, the thermoplastic starch phase greatly limited the mechanical properties and performance of the material. Consequently, improving the mechanical properties of thermoplastic starch is an effective way to improve the mechanical properties of PBAT/TPS composite materials. In previous reports, nano-inorganic powders (modified clay, nano-silica, and nano-zinc oxide) and fiber were able to significantly improve the mechanical properties of starch [32,33,34,35,36]. However, there were no reports of such modified thermoplastic starches being blended with PBAT and a detailed study was necessary. It is believed that thermoplastic starch reinforced by nano-silica can further increase the thermoplastic starch content in PBAT-based materials while maintaining serviceability. This will further reduce the production costs of PBAT/TPS composites films.

This study was conducted to improve the mechanical properties of thermoplastic starches by incorporating silica nanoparticles as reinforcing agents in the preparation of thermoplastic starches. Then PBAT was then mixed with thermoplastic hydroxypropyl starch reinforced with different nano-silica content at a ratio of 8/2 to prepare the composite film, and the mechanical properties of the composite films were characterised to select the optimum amount of nano-silica. After selecting the strongest thermoplastic hydroxypropyl starch, the PBAT-based composite films with high thermoplastic starch content were blended proportionally. The mechanical properties, thermal properties, and microscopic morphology of the films were also characterized, and high-strength, low-cost PBAT-based fully biodegradable films were obtained.

**Table 1:** Literature review on the effect of TPS content on the mechanical properties of PBAT/TPS films.

Source of Starch	Compatibilizer/ Additive	TPS Ratio in PBAT/TPS	Film Mechanical Properties		Reference
			TS	EB	
Cassava	Neat sepiolite	50	5.3	120	[37]
Corn	Maleic anhydride	10–50	4.4–13.4	256–540	[38]
Potato	Maleic anhydride PBAT-g-MA	40–50	6.2–7.9	196–220	[9]
Corn	Citric acid Tween 80	20–50	2.3–4.0	7–21	[39]
Cassava	Soybean oil Tween 80	50	0.5–6.1	32–400	[10]
Cassava	-	20–60	6.6–8.3	547–819	[40]
Cassava	Citric acid Water soluble curcumin	38	5.8–6.0	72–258	[41]
Acetylated cassava	Nisin Nisin-EDTA	40	1–14	200–950	[42]
Corn	Maleic anhydride	50	5.3	212	[13]

## 2. Materials and Methods

### 2.1. Materials

PBAT (TH801t, blown-film grade) was obtained from Blue Ridge Tunhe Science and Technology Co., Ltd. (Xinjiang, China) with a density of 1.2–1.28 g/cm^3^. Hydroxypropyl starch (HS-HP-102, Food grade) (HS) was purchased from Hengrui Starch Technology Co., Ltd. (Luohe, China) with a hydroxypropyl group content under 7.0%. Glycerol (Food grade) was obtained from Procter & Gamble (China) Co., Ltd. (Shanghai, China). Nano-silica (HL-200) was purchased from Huifu nanomaterials Co., Ltd. (Yichang, China) with hydroxyl group of 4.0–4.5% and specific surface area of 200 ± 20 m^2^/g.

### 2.2. Methods

#### 2.2.1. Preparation of Composite Film Materials

Hydroxypropyl starch (HS) was dried in an oven at 80 °C for 8 h, and nano-silica was dried at 105 °C for 2 h. Hydroxypropyl starch, glycerol, and nano-silica were mixed in a FW100 high speed mixers by Tianjin Teste Instruments Co., Ltd. (Tianjin, China) at room temperature for 20 min according to the formula of Table 2 and stored in a sealed container until use. The mixtures were compounded into TPHS_g-SiO2_ pellets by extrusion, using a KTE-20 laboratory twin-screw extruder (Nanjing Keke Extrusion Equipment Co., Ltd., Nanjing, China) with a screw diameter (D) of 21.6 mm and length of 40 D. The barrel temperatures from the feed zone to the die zone were 100, 130, 140, 145, 150, 145 °C. The screw speed was 100 rpm. The extruded strands were air-cooled, cut into pellets, and stored in a sealed container until used.

PBAT and TPHS_g-4SiO2_ were then mixed according to the formulation in Table 3 and extruded with the same laboratory twin-screw extruder. The barrel temperatures from the feed zone to the die zone were 150, 155, 160, 165, 160, 155 °C. The screw speed was 120 rpm. The extruded strands were water-cooled, cut into pellets, and dried in a vacuum oven at 80 °C for 12 h, stored in a sealed container until use. When exploring the optimum nano-silica content, the ratio of PBAT/TPHS_g-SiO2_ was 8/2 and the nano-silica content in TPHS was 1, 2, 3, 4, and 5 phr; the ratios for PBAT/TPHS_g-4SiO2_ composite films with different starch content were explored as shown in Table 2 and the nano-silica content in TPHS was 4 phr. Different percentages of PBAT/TPHS_g_ pellets were prepared in the same way.

The films were prepared using a JFYC-28 laboratory small testing film-blowing machine by Guangzhou Jinfang Yuan Machinery Manufacturing Co., Ltd. (Guangzhou, China) with a die diameter of 40 mm. The film-blowing conditions consisted of a screw speed of 15 rpm and extrusion temperature profiles of 135, 165, 155, and 140 °C, with a blow-up ratio of 2.8–3.0 and a traction ratio of 3.0. The film thickness was kept at 50–60 μm (80 ± 5 μm for samples with 50% TPHS_g_ content, 60% TPHS_g-4SiO2_ content samples cannot be blown due to the material phase structure with TPHS_g_ as a continuous phase).

#### 2.2.2. Characterization of the Composite Films

The static tensile tests were performed using a CMT6104 tensile tester by Meters Industrial Systems (China) Co., Ltd. (Shenzhen, China). The tensile strength (TS) and elongation at break (EB) of the films were evaluated according to GB/T 1040.3-2006. Film samples were cut into strips (200 mm long and 20 mm wide), with a spacing of 50 mm and a stretching rate of 200 mm/min. The thickness of the film was measured using a digital flush micrometer gauge, measuring five positions on each film, and taking the average. The tests were performed with at least ten replicates for each sample.

The thermal properties of the composite films were characterized using a Q20 differential scanning calorimeter by TA instruments (New Castle, DE, USA). An about 5–6 mg sample was weighed and placed in a crucible under nitrogen atmosphere. The test temperature was first increased from 40 °C to 180 °C, held for 5 min to eliminate the thermal history of the sample, then cooled to −60 °C, held for 5 min, and then increased to 200 °C, at a rate of 10 °C/min. The crystallization temperature (*T_c_*) and the heat of crystallization (∆*H_c_*) were obtained from the first temperature reduction, while the glass transition temperature (*T_g_*), the melting temperature (*T_m_*), and the heat of melt (∆*H_m_*) were determined in the second temperature increase, and the degree of crystallinity was calculated from the following equation.

(1) $$Xc\%=\frac{\Delta H_{c}}{\Delta H_{c}^{0}\times \left(1-a\right)}\times 100\%$$

where ∆*H_c_* is the heat of crystallization of the sample; $\Delta H_{c}^{0}$ is the heat of crystallization of pure PBAT, 70.69 J/g; a is the weight fraction of thermoplastic hydroxypropyl starch.

Note: According to the NMR test data, the mass proportion of PBT segment in the PBAT was 48.75%, and the enthalpy of crystallization of PBT segment was 145 J/g. Therefore, the heat of complete crystallization ($\Delta H_{c}^{0}$) of pure PBAT sample was 70.69 J/g.

The thermal stability properties of the PBAT/TPHS_g-SiO2_ composite film material were characterized using the thermogravimetric analyzer (TG-209, NETZSCH Scientific Instruments Trading (Shanghai) Ltd., Shanghai, China). The testing temperature program was 30 °C, heating to 800 °C with a heating rate of 10 °C/min under a nitrogen atmosphere and sample mass of 8–9 mg.

Scanning electron microscope (ZEISS Ultra 55, CarlZeiss Jena, Oberkochen, Germany) was used to observe the surface and cross-sectional morphology of the composite films. The films were frozen in liquid nitrogen. The samples were placed in water for 24 h, allowing the TPHS_g-SiO2_ phase to be completely removed from the film. The film samples were placed on the device and plated with gold using plasma sputtering for 20 min before observation.

The PBAT/TPHS_g-SiO2_ composites were tested for the melt flow index according to GB/T 3682-2000. The tests were carried out at 150 °C and 190 °C, respectively, with a weight of 2160 g. The melt flow index values in g/10 min were calculated using Equation (2).

(2) $$MI=\frac{t_{ref}\cdot m}{t}$$

where: $t_{ref}$ is the reference time, s (600 s); *m* is the average mass of cut sections, g; *t* is the time interval between cut sections, s.

Data in this work was expressed as the mean along with its corresponding standard deviation (SD). The analysis of variance (ANOVA) was performed with SPSS (version 27.0 for windows, IBM, New York, NY, USA) and the Tukey test was used to determine significant difference between means (*p* < 0.05).

## 3. Results

### 3.1. Mechanical Properties

**Figure 1:**
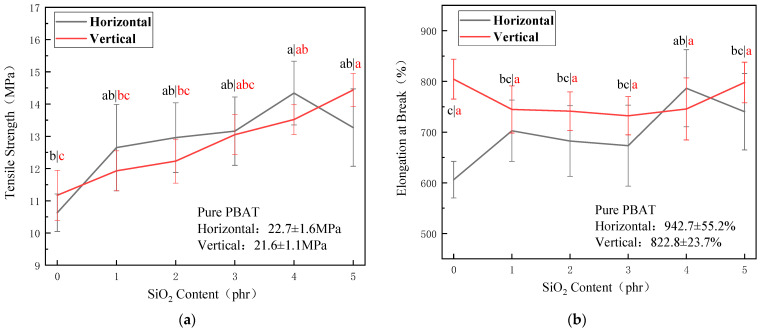
Effect of SiO_2_ dosage on mechanical properties of PBAT/TPHS_g-SiO2_ (8/2) composite films. (**a**) Tensile strength; (**b**) Elongation at break.

**Figure 2:**
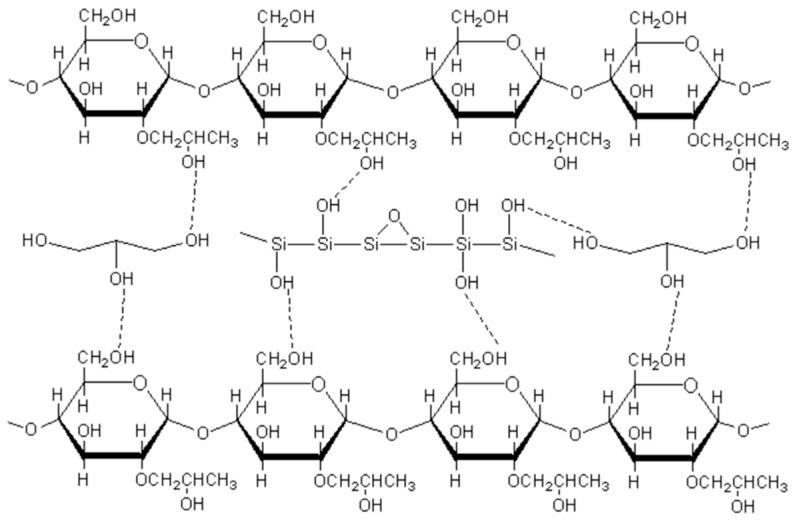
Nano-silica reinforced plasticized hydroxypropyl starch.

Figure 1 shows the tensile strength (Figure 1a) and elongation at break (Figure 1b) of the composite films prepared by PBAT with 20% of thermoplastic hydroxypropyl starch with different nano-silica contents. The horizontal and vertical tensile strengths of the film tend to increase with the amount of nano-silica in the thermoplastic hydroxypropyl starch, reaching a maximum of 4 phr of nano-silica. Compared to the sample without silica, the horizontal tensile strength of the film increased from 10.63 MPa to 14.34 MPa, with an increase of 35%, and the vertical tensile strength increased from 11.17 MPa to 13.52 MPa, with an increase of 21%. Elongation at break of the film was between 600 and 800%. The increase in the tensile strength of the composite film is mainly due to the increased mechanical strength of the thermoplastic hydroxypropyl starch, which effectively disperses the stress during film stretching and thus increases the overall tensile strength of the film. According to the findings of Zhu et al. [33], nano-silica has been shown to enhance hydroxypropyl starch by the mechanism shown in Figure 2, mainly due to the interfacial adhesion between the starch and the SiO_2_ nanoparticles, which is caused by the C-O-Si chemical bond, the formation of hydrogen bonds between the silicon hydroxyl groups on the surface of the silica nanoparticles, and the large number of silicon hydroxyl groups in the starch matrix [43].

**Figure 3:**
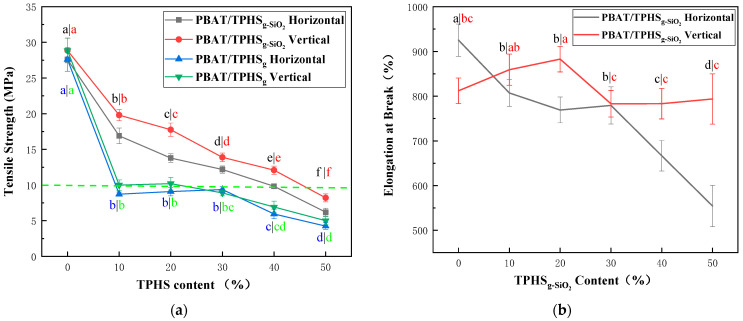
Effect of TPHS content on mechanical properties of PBAT/TPHS_g-4SiO2_ composite films. (**a**) Tensile strength; (**b**) Elongation at break.

Figure 3a shows the tensile strength of PBAT/TPHS_g-4SiO2_ and PBAT/TPHS_g_ composite films with different TPHS content. As the content of TPHS in the film increases, the tensile strength of the film gradually decreases, and the vertical tensile strength of the composite film is slightly higher than the horizontal tensile strength. The horizontal and vertical tensile strength of the PBAT/TPHS_g-4SiO2_ composite film is significantly higher than that of the PBAT/TPHS_g_ composite film with the same amount of thermoplastic starch. It shows that the reinforcement of thermoplastic hydroxypropyl starch by nano-silica is an effective way to improve the tensile strength of PBAT/TPHS composite films. At a TPHS_g-4SiO2_ content of 40%, the film also maintains horizontal and vertical tensile strengths of 9.82 MPa and 12.09 MPa, both of which meet the requirements for commercial films, according to the relevant standards, such as GB/T 18893-2002, GB/T 4456-1996, and GB/T 38082-2019. When the content of TPHS_g-4SiO2_ is 50%, the horizontal and vertical tensile strengths of the film are 6.20 MPa and 8.21 MPa, respectively, which can still meet the mechanical property requirements of agricultural film materials, according to the relevant standards, such as GB/T 35795-2017, showing a good prospect of use in the field of fully biodegradable agricultural ground cover films.

**Figure 4:**
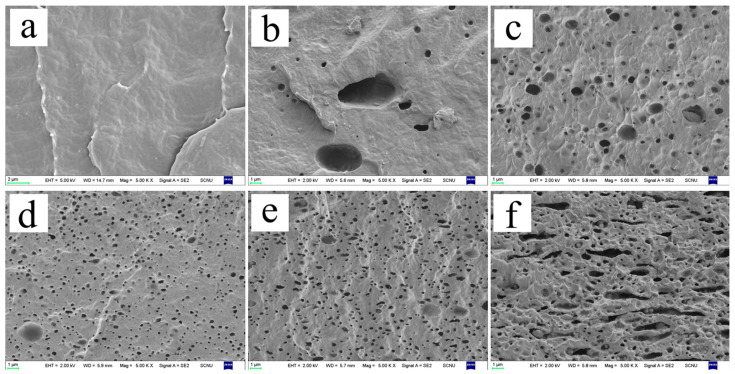
Scanning electron micrographs of the cross-section of PBAT and composite films (5000×). (**a**) pure PBAT 5000×; (**b**) 90PBAT/10TPHS_g-4SiO2_ 5000×; (**c**) 80PBAT/20TPHS_g-4SiO2_ 5000×; (**d**) 70PBAT/30TPHS_g-4SiO2_ 5000×; (**e**) 60PBAT/40TPHS_g-4SiO2_ 5000×; (**f**) 50PBAT/50TPHS_g-4SiO2_ 5000×.

Figure 3b shows the change in elongation at break for the composite films. The vertical elongation at break of the composite films ranged from 800 to 900%, which is similar to the 812% of pure PBAT. After adding TPHS_g-4SiO2_ as a filler to reduce costs, the PBAT/TPHS_g-4SiO2_ composite film maintains a good elongation at break, avoiding the disadvantages of thermoplastic starches with low elongation at break and brittle materials. However, the horizontal elongation at break of the film has a clear tendency to decrease as the TPHS_g-4SiO2_ content increases, and the rate of decrease increases significantly after the TPHS_g-4SiO2_ content reaches 40%, decreasing to 666% at 40% TPHS_g-4SiO2_ content and 554% at 50% TPHS_g-4SiO2_ content, 40.1% reduction compared to PBAT films. The reason for the drastic decrease in elongation at break may be that the PBAT/TPHS_g-4SiO2_ composite film changed from an island structure to a bi-continuous phase structure (as shown in Figure 4e), the PBAT and TPHS_g-4SiO2_ phases were oriented in the vertical direction during the blowing of the film, and the PBAT phase was no longer continuous in the horizontal direction of the film. Since the mechanical strength of the thermoplastic starch phase is much lower than the PBAT phase, when the bi-continuous phase structure appears, the composite film easily breaks during stretching due to defects in the starch phase, which is also the reason why the tensile strength decreases when the TPHS_g-4SiO2_ content increases.

In addition, the mechanical properties of PBAT/TPHS_g-4SiO2_ composite films are comparable to LDPE materials which are commonly used in packaging and agriculture. The thermoplastic starch content of 50% still has good mechanical properties, PBAT improves the mechanical properties and flexibility of the thermoplastic starch. Zhai et al. [44] reported PBAT/TPS composite films prepared with glycerol and citric acid as plasticizers and nano-clay as reinforcing agent. The horizontal and vertical tensile strengths of the films at 50% of thermoplastic starch were 5.3 and 7.4 MPa. PBAT/TPS composite films with a thermoplastic starch content of 50% were prepared by Pan et al. [38] The horizontal and vertical tensile strengths were 4.4/4.6 MPa, and the films had 256/277% elongation at break in the horizontal and vertical directions. In this work, the mechanical properties of PBAT/TPHS_g-4SiO2_ composite films were improved by adding nano-silica as reinforcing agent for thermoplastic hydroxypropyl starch.

### 3.2. Melt Flow Index

Table 4 shows the melt flow index of PBAT and thermoplastic hydroxypropyl starch composites. The melt flow index of the material increases and then decreases with the increase in TPHS_g-4SiO2_ phase content, and the MFR is maximum at the TPHS_g-4SiO2_ content of 20%. The reason may be that at low TPHS_g-4SiO2_ content, the small molecules of glycerol in the TPHS_g-4SiO2_ transferred to PBAT and the melting index of the composite increases. When the TPHS_g-4SiO2_ content reaches 30%, the phase structure of the composites is transformed. As shown in the SEM results, the phase structure is changed into a two-phase continuous structure at a high TPHS_g-4SiO2_ content, and the high viscosity of the TPHS_g-4SiO2_ phase itself restricts the flow of the composites, which reduces the fluidity of the composites and leads to the reduction of the melt index of the composites. When the content of TPHS_g-4SiO2_ reaches 60%, it does not have the ability to blow film at 140–160 °C blowing temperature because the flowability is poor, and it has been cooled at the die.

### 3.3. Scanning Electron Microscope

Figure 4 shows the cross-sectional morphology of the composite films. The contents of thermoplastic hydroxypropyl starch in Figure 4a–f are 0%, 10%, 20%, 30%, 40%, and 50%, respectively. The film sections were freeze-fragmentations in liquid nitrogen and dipped in distilled water for one day to remove the starch phase from the film completely. Figure 4a shows the cross-sectional morphology of pure PBAT with smooth cross-section. As the content of TPHS_g-4SiO2_ gradually increased, the number of holes increased significantly, and the holes show spherical or sphere-like shapes. When the content of TPHS_g-4SiO2_ was 40% and above, the TPHS_g-4SiO2_ balls were deformed and connected, and the phase structure of the composites changed from island structures to two-phase continuous structures. This also leads to a dramatic decrease in mechanical strength and decrease in elongation at break of the film in the horizontal direction.

When the TPHS_g-4SiO2_ content varied from 10% to 30%, electron micrographs show the thermoplastic starch microspheres changing from 3 μm to smaller than 0.5 μm. Due to the strong hydrogen bonding of the starch and the lower viscosity of PBAT, the TPHS_g-4SiO2_ spheres cannot disperse easily, so the diameter of TPHS_g-4SiO2_ spheres is larger. When the TPHS_g-4SiO2_ content increased, the TPHS_g-4SiO2_ spheres squeeze each other and break, making the diameter of the spheres smaller.

**Figure 5:**
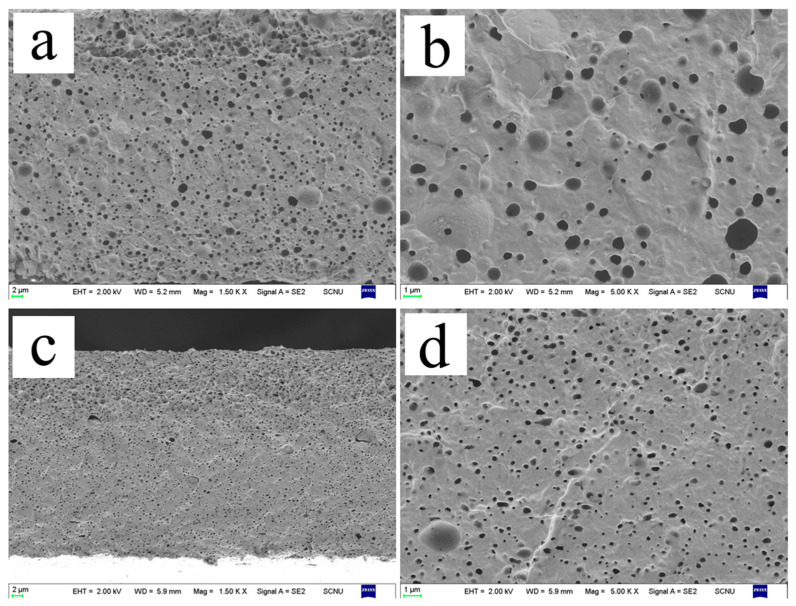
Comparison of the cross-sectional morphology of PBAT-based composite films of two thermoplastic starches. (**a**) 70PBAT/30TPHS_g_ 1500×; (**b**) 70PBAT/30TPHS_g_ 5000×; (**c**) 70PBAT/30TPHS_g-4SiO2_ 1500×; (**d**) 70PBAT/30TPHS_g-4SiO2_ 5000×.

Figure 5 shows a comparison of the cross-sectional morphology of both 70PBAT/30TPHS_g_ and 70PBAT/30TPHS_g-4SiO2_ composite films. It is clear that the number of holes of the 70PBAT/30TPHS_g_ composite film is fewer than the one observed for the 70PBAT/30TPHS_g-4SiO2_ composite film, but the diameter of the holes in the former is significantly larger than the latter. This is due to the thermoplastic starch having a higher melt strength after reinforcement with nano-silica. During the melt blending process, the interaction between thermoplastic starch pellets is more significant, resulting in the fragmentation of the starch pellets. Therefore, the reinforced thermoplastic starch is better dispersed in PBAT. Better dispersion not only allows the PBAT phase to maintain continuity, but also prevents the breakage of starch granules during the stretching process, which can lead to defects and film breakage. This is the same result as the tensile strength of the PBAT/TPHS_g-4SiO2_ composite film was higher than that of the PBAT/TPHS_g_ composite film.

### 3.4. Differential Scanning Calorimetry

**Figure 6:**
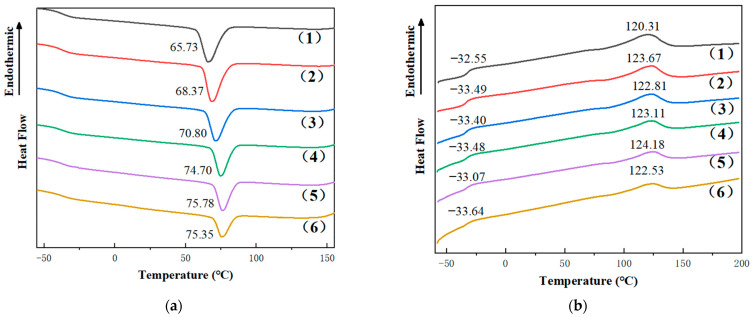
Changes in the DSC curves of PBAT/TPHS_g-4SiO2_ composite films with the addition of TPHS_g-4SiO2_. (**a**) The first cooling process; (**b**) the second heating process. (1) Black: Pure PBAT; (2) Red: 90PBAT/10TPHS_g-4SiO2_; (3) Blue: 80PBAT/20TPHS_g-4SiO2_; (4) Green: 70PBAT/30TPHS_g-4SiO2_; (5) Purple: 60PBAT/40TPHS_g-4SiO2_; (6) Brown: 50PBAT/50TPHS_g-4SiO2_.

Differential scanning calorimetric analyses were performed on PBAT and PBAT/TPHS_g-4SiO2_ composites to obtain the crystallization temperature, glass transition temperature, melting temperature, enthalpy of crystallization, enthalpy of melting, and crystallinity of the composites. Figure 6a shows the process of the first temperature reduction. The T_c_ temperature of pure PBAT was 65.73 °C. The crystallization temperature increased after the addition of TPHS_g-4SiO2_ and gradually increased as the content of TPHS_g-4SiO2_ increased. This is due to the TPHS_g-4SiO2_ acting as a nucleating agent and promoting the crystallization of PBAT. And as the TPHS_g-4SiO2_ content increases, the crystallization enthalpy of the composite film material gradually decreases. Chen et al. [45] demonstrated that the reduced crystallinity of polyester was beneficial to the degradation of the composite film. No crystallization process of TPHS_g-4SiO2_ was observed during the tests.

Figure 6b shows the second temperature rise process, which obverses the glass transition temperature and the melting temperature of the material. The glass transition temperature of the PBAT phase was reduced with the addition of TPHS_g-4SiO2_. It is possible that the transfer of the glycerol from TPHS_g-4SiO2_ into the PBAT phase increases the mobility of the PBAT molecular chain segments and decreases the glass transition temperature. It can also be seen that the melting points of the composites are all higher than pure PBAT. This is mainly due to the fact that the composites crystallize at higher temperatures, where the molecular chain segments are more mobile, have enough time to adjust, and the crystals are more perfect. However, the melting temperature of the composites did not change much and the crystallinity of PBAT did not change. As can be seen in Table 5, the crystallinity of the composite film material gradually decreases as the TPHS_g-4SiO2_ content increases. This is due to the increase in TPHS_g-4SiO2_ content, the increase in the crystallization sites of PBAT in the material, the faster crystallization of the PBAT phase, and the decrease in crystallinity.

### 3.5. Thermogravimetric Analysis

**Figure 7:**
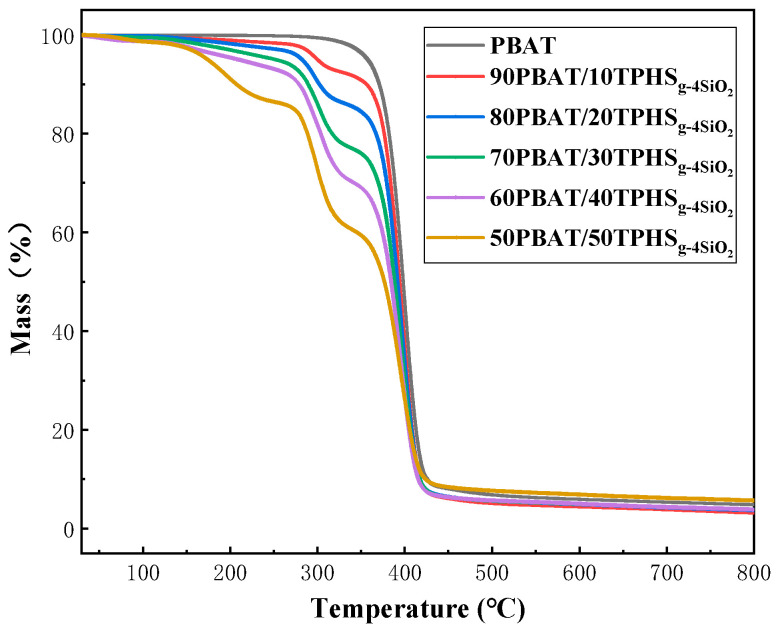
TG curves of pure PBAT and PBAT/TPHS_g-4SiO2_ composite films.

**Figure 8:**
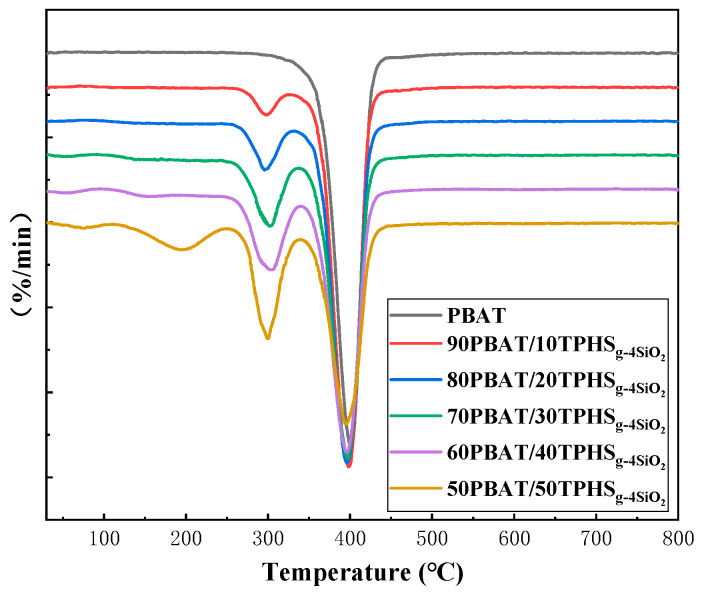
DTG curves of pure PBAT and PBAT/TPHS_g-4SiO2_ composite films.

Figure 7 and Table 6 show the TG curves of pure PBAT and PBAT/TPHS_g-4SiO2_ composite film materials and Figure 8 shows the DTG curves of pure PBAT and PBAT/TPHS_g-4SiO2_ composite film materials. In Figure 8, it can be seen that there is a first weight loss step from 150 to 260 °C, which is a crystalline water weight loss step of the TPHS_g-4SiO2_ phase and increases as the TPHS_g-4SiO2_ content increases. When the content of TPHS_g-4SiO2_ is 50%, the weight loss step increases significantly, caused firstly by the increase in water of crystallization due to the increased TPHS content, and secondly by the shift in the phase structure of the material. The transformation from island structures to two-phase continuous structures is shown in the SEM characterization results. At low TPHS_g-4SiO2_ content, the crystalline water from TPHS_g-4SiO2_ needs to pass through the PBAT phase. However, with the increase of the TPHS_g-4SiO2_ content, the precipitation of the crystalline water no longer needs to pass through the PBAT phase and the precipitation capacity of the crystalline water increases, resulting in a significantly larger weight loss step for the crystalline water.

The decomposition onset temperature of pure TPHS_g-4SiO2_ was 275.4 °C. After compounding with PBAT, the decomposition temperature of the starch phase increased and gradually decreased as the TPHS_g-4SiO2_ content increased. The decomposition peak of the starch phase gradually increases with the increase of the TPHS_g-4SiO2_ content, as shown in Figure 8. The decomposition onset temperature of pure PBAT was 400.7 °C. The addition of TPHS_g-4SiO2_ to the mix decreased the onset decomposition temperature of PBAT and showed a decreasing trend with the increase of the TPHS_g-4SiO2_ content.

## 4. Conclusions

The blend of thermoplastic hydroxypropyl starch and PBAT with the addition of silica as reinforcing agent exhibits good mechanical properties, with a substantial increase in mechanical properties compared to the sample without the addition of nano-silica. Even with a thermoplastic hydroxypropyl starch content of 40%, the film has horizontal and vertical tensile strengths of 9.82 and 12.09 MPa, respectively, which meet the requirements for packaging film. Furthermore, at thermoplastic hydroxypropyl starch levels below 50%, the processing properties of the composites are similar to those of PBAT, enabling the preparation of composite films by blowing. The micrographs also show that the thermoplastic hydroxypropyl starch is homogeneously distributed in PBAT, while the thermoplastic hydroxypropyl starch which was reinforced by nano-silica is better dispersed in PBAT, and that the content below 40% does not affect the continuous structure of the PBAT phase. Therefore, it is feasible to enhance the TPHS_g-4SiO2_ phase in PBAT/TPHS_g-4SiO2_ composite films by adding nano-silica, thus enabling the enhancement of the mechanical properties of the composite films, and the color of the composite films will be whiter after the addition of nano-silica. Our study provides an effective way to prepare PBAT-based composite films with high starch content.

## Figures and Tables

**Table 2:** Formulation of silica-enhanced thermoplastic hydroxypropyl starch.

Sample Name	HS (phr)	Glycerol (phr)	Nano-Silica (phr)
TPHS_g-0SiO2_	100	40	0
TPHS_g-1SiO2_	100	40	1
TPHS_g-2SiO2_	100	40	2
TPHS_g-3SiO2_	100	40	3
TPHS_g-4SiO2_	100	40	4
TPHS_g-5SiO2_	100	40	5

phr is weight parts.

**Table 3:** The ratio of PBAT/TPHS_g-4SiO2_ composite films.

Sample Name	PBAT (%)	TPHS_g-4SiO2_ (%)
90PBAT/10TPHS_g-4SiO2_	90	10
80PBAT/20TPHS_g-4SiO2_	80	20
70PBAT/30TPHS_g-4SiO2_	70	30
60PBAT/40TPHS_g-4SiO2_	60	40
50PBAT/50TPHS_g-4SiO2_	50	50
40PBAT/60TPHS_g-4SiO2_	40	60

**Table 4:** MI of PBAT and TPHS_g-4SiO2_ composite films.

Sample Name	Melt Flow Index (g/10 min)	
	150 °C, 2160 g	190 °C, 2160 g
PBAT	2.66 ± 0.03 ^c^	9.47 ± 0.31 ^d^
90PBAT/10TPHS_g-4SiO2_	2.85 ± 0.04 ^b^	10.89 ± 0.18 ^c^
80PBAT/20TPHS_g-4SiO2_	3.18 ± 0.08 ^a^	14.21 ± 0.40 ^a^
70PBAT/30TPHS_g-4SiO2_	2.59 ± 0.03 ^c^	12.67 ± 0.32 ^b^
60PBAT/40TPHS_g-4SiO2_	2.01 ± 0.05 ^d^	10.34 ± 0.26 ^c^
50PBAT/50TPHS_g-4SiO2_	1.09 ± 0.04 ^e^	7.60 ± 0.19 ^e^
40PBAT/60TPHS_g-4SiO2_	0.24 ± 0.02 ^f^	3.72 ± 0.15 ^f^

Each value is expressed as mean ± SD (*n* = 5). Different superscript letters in each vertical column denote statistically difference (*p* < 0.05).

**Table 5:** DSC data of pure PBAT and PBAT/TPHS_g-4SiO2_ composite films.

Sample Name	T_g_ (°C)	T_c_ (°C)	T_m_ (°C)	△H_c_ (J/g)	△H_m_ (J/g)	Crystallinity (%)
PBAT	−32.55	65.73	120.31	16.73	15.39	23.67
90PBAT/10TPHS_g-4SiO2_	−33.49	68.37	123.67	15.61	12.31	24.54
80PBAT/20TPHS_g-4SiO2_	−33.40	70.80	122.81	13.90	11.02	24.58
70PBAT/30TPHS_g-4SiO2_	−33.48	74.70	123.11	11.31	8.16	22.86
60PBAT/40TPHS_g-4SiO2_	−33.07	75.78	124.18	9.56	6.91	22.54
50PBAT/50TPHS_g-4SiO2_	−33.64	75.35	122.53	6.72	5.89	19.02

**Table 6:** TG data of pure PBAT and PBAT/TPHS_g-4SiO2_ composite films.

Sample Name	T_90%_ (°C)	T_50%_ (°C)	Residual Rate (%)	T_start_ (°C)		T_max_ (°C)	
				Step 1	Step 2	Step 1	Step 2
PBAT	370.9	398.3	4.85	/	377.6	/	400.7
TPHS_g-4SiO2_	261.7	306.5	11.35	275.4	/	304.2	/
90PBAT-10TPHS_g-4SiO2_	356.2	394.3	3.19	286.4	377.3	298.0	398.2
80PBAT-20TPHS_g-4SiO2_	302.7	392.0	3.61	285.9	376.4	295.7	396.2
70PBAT-30TPHS_g-4SiO2_	288.9	388.4	3.97	286.2	376.0	302.0	396.8
60PBAT-40TPHS_g-4SiO2_	278.9	384.4	3.88	283.4	374.9	304.7	396.5
50PBAT-50TPHS_g-4SiO2_	206.8	377.0	5.70	279.6	375.1	314.9	394.6

## Data Availability

All data included in this study are available upon request to the corresponding author.

## References

[1] Shah A.A., Kato S., Shintani N., Kamini N.R., Nakajima-Kambe T. (2014). Microbial degradation of aliphatic and aliphatic-aromatic co-polyesters. Appl. Microbiol. Biotechnol..

[2] Leal I.L., da Silva Rosa Y.C., da Silva Penha J., Correia P.R.C., da Silva Melo P., Guimarães D.H., Barbosa J.D.V., Druzian J.I., Machado B.A.S. (2019). Development and application starch films: PBAT with additives for evaluating the shelf life of Tommy Atkins mango in the fresh-cut state. J. Appl. Polym. Sci..

[3] Youssef A.M., Assem F.M., Abdel-Aziz M.E., Elaaser M., Ibrahim O.A., Mahmoud M., Abd El-Salam M.H. (2019). Development of bionanocomposite materials and its use in coating of Ras cheese. Food Chem..

[4] Kale G. (2011). Overview of Biodegradable Packaging, Methods, and Current Trends.

[5] Bittmann B., Bouza R., Barral L., González-Rodríguez M.V., Abad M.-J. (2012). Nanoclay-reinforced poly(butylene adipate-co-terephthalate) biocomposites for packaging applications. Polym. Compos..

[6] Lai L., Wang S., Li J., Liu P., Wu L., Wu H., Xu J., Severtson S.J., Wang W.J. (2020). Stiffening, strengthening, and toughening of biodegradable poly(butylene adipate-co-terephthalate) with a low nanoinclusion usage. Carbohydr. Polym..

[7] Ferreira F.V., Mariano M., Pinheiro I.F., Cazalini E.M., Souza D.H.S., Lepesqueur L.S.S., Koga-Ito C.Y., Gouveia R.F., Lona L.M.F. (2019). Cellulose nanocrystal-based poly(butylene adipate-co-terephthalate) nanocomposites covered with antimicrobial silver thin films. Polym. Eng. Sci..

[8] Wang H.T., Wang J.M., Wu T.M. (2019). Synthesis and characterization of biodegradable aliphatic–aromatic nanocomposites fabricated using maleic acid-grafted poly[(butylene adipate)-co-terephthalate] and organically modified layered zinc phenylphosphonate. Polym. Int..

[9] Dammak M., Fourati Y., Tarrés Q., Delgado-Aguilar M., Mutjé P., Boufi S. (2020). Blends of PBAT with plasticized starch for packaging applications: Mechanical properties, rheological behaviour and biodegradability. Ind. Crops Prod..

[10] Brandelero R.P.H., Grossmann M.V., Yamashita F. (2012). Films of starch and poly(butylene adipate co-terephthalate) added of soybean oil (SO) and Tween 80. Carbohydr. Polym..

[11] Olivato J.B., Grossmann M.V.E., Bilck A.P., Yamashita F. (2012). Effect of organic acids as additives on the performance of thermoplastic starch/polyester blown films. Carbohydr. Polym..

[12] Wei D., Wang H., Xiao H., Zheng A., Yang Y. (2015). Morphology and mechanical properties of poly(butylene adipate-co-terephthalate)/potato starch blends in the presence of synthesized reactive compatibilizer or modified poly(butylene adipate-co-terephthalate). Carbohydr. Polym..

[13] Chang C.C., Trinh B.M., Mekonnen T.H. (2021). Robust multiphase and multilayer starch/polymer (TPS/PBAT) film with simultaneous oxygen/moisture barrier properties. J. Colloid Interface Sci..

[14] Fourati Y., Tarres Q., Mutje P., Boufi S. (2018). PBAT/thermoplastic starch blends: Effect of compatibilizers on the rheological, mechanical and morphological properties. Carbohydr. Polym..

[15] Liu Y., Liu S., Liu Z., Lei Y., Jiang S., Zhang K., Yan W., Qin J., He M., Qin S. (2021). Enhanced mechanical and biodegradable properties of PBAT/lignin composites via silane grafting and reactive extrusion. Compos. Part B Eng..

[16] Kargarzadeh H., Galeski A., Pawlak A. (2020). PBAT green composites: Effects of kraft lignin particles on the morphological, thermal, crystalline, macro and micromechanical properties. Polymer.

[17] Xie X., Zhang C., Weng Y., Diao X., Song X. (2020). Effect of Diisocyanates as Compatibilizer on the Properties of BF/PBAT Composites by In Situ Reactive Compatibilization, Crosslinking and Chain Extension. Materials.

[18] Xu Z., Qiao X., Sun K. (2020). Environmental-friendly corn stover/poly(butylene adipate-co-terephthalate) biocomposites. Mater. Today Commun..

[19] Borges J.A., Romani V.P., Cortez-Vega W.R., Martins V.G. (2015). Influence of different starch sources and plasticizers on properties of biodegradable films. Int. Food Res. J..

[20] Nunes M.A.B.S., Marinho V.A.D., Falcão G.A.M., Canedo E.L., Bardi M.A.G., Carvalho L.H. (2018). Rheological, mechanical and morphological properties of poly(butylene adipate-co-terephthalate)/thermoplastic starch blends and its biocomposite with babassu mesocarp. Polym. Test..

[21] Biliaderis C.G., Maurice T.J., Vose J.R. (1980). Starch Gelatinization Phenomena Studied by Differential Scanning Calorimetry. J. Food Sci..

[22] Lefevre C., Bohuon P., Akissoe L., Ollier L., Matignon B., Mestres C. (2021). Modeling the gelatinization-melting transition of the starch-water system in pulses (lentil, bean and chickpea). Carbohydr. Polym..

[23] Wang J., Liang Y., Zhang Z., Ye C., Chen Y., Wei P., Wang Y., Xia Y. (2021). Thermoplastic starch plasticized by polymeric ionic liquid. Eur. Polym. J..

[24] Wadaugsorn K., Panrong T., Wongphan P., Harnkarnsujarit N. (2022). Plasticized hydroxypropyl cassava starch blended PBAT for improved clarity blown films: Morphology and properties. Ind. Crops Prod..

[25] Asnawi T.M., Zaki M., Khadafi M., Harmanita I. (2022). Synthesis and characterization of biodegradable plastic from watermelon rind starch and chitosan by using glycerol as plasticizer. Mater. Today Proc..

[26] Yu F., Prashantha K., Soulestin J., Lacrampe M.F., Krawczak P. (2013). Plasticized-starch/poly(ethylene oxide) blends prepared by extrusion. Carbohydr. Polym..

[27] Lerma-Canto A., Gomez-Caturla J., Herrero-Herrero M., Garcia-Garcia D., Fombuena V. (2021). Development of Polylactic Acid Thermoplastic Starch Formulations Using Maleinized Hemp Oil as Biobased Plasticizer. Polymers.

[28] Momeni S., Rezvani Ghomi E., Shakiba M., Shafiei-Navid S., Abdouss M., Bigham A., Khosravi F., Ahmadi Z., Faraji M., Abdouss H. (2021). The Effect of Poly (Ethylene glycol) Emulation on the Degradation of PLA/Starch Composites. Polymers.

[29] Bidari R., Abdillah A.A., Ponce R.A.B., Charles A.L. (2023). Characterization of Biodegradable Films Made from Taro Peel (Colocasia esculenta) Starch. Polymers.

[30] Hazrol M.D., Sapuan S.M., Zainudin E.S., Zuhri M.Y.M., Abdul Wahab N.I. (2021). Corn Starch (*Zea mays*) Biopolymer Plastic Reaction in Combination with Sorbitol and Glycerol. Polymers.

[31] Mina Hernandez J.H. (2020). Effect of the Incorporation of Polycaprolactone (PCL) on the Retrogradation of Binary Blends with Cassava Thermoplastic Starch (TPS). Polymers.

[32] Gao W., Dong H., Hou H., Zhang H. (2012). Effects of clays with various hydrophilicities on properties of starch–clay nanocomposites by film blowing. Carbohydr. Polym..

[33] Zhu J., Gao W., Wang B., Kang X., Liu P., Cui B., Abd El-Aty A.M. (2021). Preparation and evaluation of starch-based extrusion-blown nanocomposite films incorporated with nano-ZnO and nano-SiO(2). Int. J. Biol. Macromol..

[34] López J.P., Mutjé P., Carvalho A.J.F., Curvelo A.A.S., Gironès J. (2013). Newspaper fiber-reinforced thermoplastic starch biocomposites obtained by melt processing: Evaluation of the mechanical, thermal and water sorption properties. Ind. Crops Prod..

[35] Gironès J., López J.P., Mutjé P., Carvalho A.J.F., Curvelo A.A.S., Vilaseca F. (2012). Natural fiber-reinforced thermoplastic starch composites obtained by melt processing. Compos. Sci. Technol..

[36] López O.V., Castillo L.A., García M.A., Villar M.A., Barbosa S.E. (2015). Food packaging bags based on thermoplastic corn starch reinforced with talc nanoparticles. Food Hydrocoll..

[37] Olivato J.B., Marini J., Pollet E., Yamashita F., Grossmann M.V., Averous L. (2015). Elaboration, morphology and properties of starch/polyester nano-biocomposites based on sepiolite clay. Carbohydr. Polym..

[38] Pan H., Ju D., Zhao Y., Wang Z., Yang H., Zhang H., Dong L. (2016). Mechanical properties, hydrophobic properties and thermal stability of the biodegradable poly(butylene adipate-co-terephthalate)/maleated thermoplastic starch blown films. Fibers Polym..

[39] Ivanič F., Kováčová M., Chodák I. (2019). The effect of plasticizer selection on properties of blends poly(butylene adipate-co-terephthalate) with thermoplastic starch. Eur. Polym. J..

[40] Garalde R.A., Thipmanee R., Jariyasakoolroj P., Sane A. (2019). The effects of blend ratio and storage time on thermoplastic starch/poly(butylene adipate-co-terephthalate) films. Heliyon.

[41] Mücke N., da Silva T.B.V., de Oliveira A., Moreira T.F.M., Venancio C.D.S., Marques L.L.M., Valderrama P., Gonçalves O.H., da Silva-Buzanello R.A., Yamashita F. (2021). Use of Water-Soluble Curcumin in TPS/PBAT Packaging Material: Interference on Reactive Extrusion and Oxidative Stability of Chia Oil. Food Bioprocess Technol..

[42] Leelaphiwat P., Pechprankan C., Siripho P., Bumbudsanpharoke N., Harnkarnsujarit N. (2022). Effects of nisin and EDTA on morphology and properties of thermoplastic starch and PBAT biodegradable films for meat packaging. Food Chem..

[43] Tang H., Xiong H., Tang S., Zou P. (2009). A starch-based biodegradable film modified by nano silicon dioxide. J. Appl. Polym. Sci..

[44] Zhai X., Wang W., Zhang H., Dai Y., Dong H., Hou H. (2020). Effects of high starch content on the physicochemical properties of starch/PBAT nanocomposite films prepared by extrusion blowing. Carbohydr. Polym..

[45] Chen M., Cai C., Bao J., Du Y., Gao H., Liu X. (2022). Effect of aliphatic segment length and content on crystallization and biodegradation properties of aliphatic-aromatic co-polyesters. Polym. Degrad. Stab..

